# Transplanted Human Umbilical Cord Mesenchymal Stem Cells Facilitate Lesion Repair in B6.Fas Mice

**DOI:** 10.1155/2014/530501

**Published:** 2014-12-29

**Authors:** Guang-ping Ruan, Xiang Yao, Shuang-juan Yang, Jin-xiang Wang, Fan Shu, Zi-an Li, Ju-fen Liu, Rong-qing Pang, Xing-hua Pan

**Affiliations:** ^1^Stem Cell Engineering Laboratory of Yunnan Province, Kunming General Hospital of Chengdu Military Command, Kunming 650032, China; ^2^Kunming Biological Diversity Regional Center of Large Apparatus and Equipment, Kunming Institute of Zoology, Chinese Academy of Sciences, Kunming 650223, China

## Abstract

*Background*. Systemic lupus erythematosus (SLE) is a multisystem disease that is characterized by the appearance of serum autoantibodies. No effective treatment for SLE currently exists. *Methods*. We used human umbilical cord mesenchymal stem cell (H-UC-MSC) transplantation to treat B6.Fas mice. *Results*. After four rounds of cell transplantation, we observed a statistically significant decrease in the levels of mouse anti-nuclear, anti-histone, and anti-double-stranded DNA antibodies in transplanted mice compared with controls. The percentage of CD4^+^CD25^+^Foxp3^+^ T cells in mouse peripheral blood significantly increased after H-UC-MSC transplantation. *Conclusions*. The results showed that H-UC-MSCs could repair lesions in B6.Fas mice such that all of the relevant disease indicators in B6.Fas mice were restored to the levels observed in normal C57BL/6 mice.

## 1. Introduction

Systemic lupus erythematosus (SLE) is a multisystem autoimmune disease. SLE was generally fatal before the advent of immunosuppressive medications. However, despite these advances in immunosuppressive medical therapies, SLE remains potentially fatal in some patients, particularly in those patients with treatment-refractory disease.

SLE is characterized by antibodies associated with injuries to multiple organs of the renal, cardiovascular, neural, musculoskeletal, and cutaneous systems [1]. The pathology of SLE involves the destruction of targeted organ tissues and the accumulation of autoreactive lymphocytes and immune complexes. Although disease severity and organ involvement vary significantly among SLE patients, T and B lymphocyte abnormalities are universal [1–3]. Moreover, SLE manifests multifaceted immune modulation that induces both deficiency and hyperactivity of the immune system. A deeper understanding of the underlying pathology is crucial for developing optimal therapies to restore immune homeostasis without compromising the protective immune response to pathogens [4].

In addition to conventional medical therapies, such as cyclophosphamide (CTX) and mycophenolate mofetil, several new strategies have been developed that target specific pathways relevant to SLE pathogenesis [1, 5]. For example, B cell depletion therapies using the monoclonal antibodies rituximab and epratuzumab have benefited a specific subpopulation of SLE patients [6]. Recently, hematopoietic stem cell transplantation has been reported to improve disease severity in treatment-refractory SLE patients [7] and to reverse organ dysfunction in several animal models [8]. Despite improved supportive care, aggressive immunosuppressive medical therapies, and new therapeutic interventions, certain SLE patients continue to suffer significant morbidity and mortality resulting from active disease with visceral organ involvement. Therefore, the development of more effective therapies for SLE, particularly for treatment-refractory patients, is urgently needed.

SLE is a multisystem disease that is characterized by the appearance of serum autoantibodies [9]. Treatment with glucocorticoids and immunosuppressive drugs can also have negative consequences, including bone marrow suppression and an increased rate of infection. While no effective treatment for SLE currently exists, in the past 10 years, considerable experimental and clinical evidence has indicated that stem cells, including bone marrow mesenchymal stem cells (BM-MSCs) and umbilical cord MSCs (UC-MSCs), have significant roles in cell and tissue repair, fibrosis suppression, immune suppression, and immune regulation. The biological roles of MSCs suggest their potential use in the treatment of SLE.

MSCs are derived from the mesoderm during early development and display the high self-renewal and differentiation potential of pluripotent stem cells [10]. While the most common source of MSCs is the bone marrow, MSCs are also available from other tissues, including peripheral blood, umbilical cord blood, bone tissue, cartilage tissue, muscle tissue, tendon tissue, adipose tissue, and vascular tissue. Compared with the bone marrow, umbilical cord blood is an ample source of poorly immunogenic MSCs that are able to tolerate human leukocyte antigen (HLA) mismatches to a greater degree, leading to a lower incidence of graft-versus-host disease (GVHD). For this reason and others, H-UC-MSCs are a primary source of clinical MSCs. In 2000, Erices et al. [11] first reported that MSCs could be isolated and cultured from cord blood. H-UC-MSC isolation and culture methods were established by work that was completed by Romanov et al. [12].

Because they have the capacity to differentiate into various cell types, including osteoblasts, chondrocytes, adipocytes, muscle (tendon) cells, liver cells, and nerve cells [13], MSCs are potentially capable of repairing the multisystem and multiorgan damage resulting from SLE. H-UC-MSCs were able to treat SLE successfully in humans. Therefore, we investigated whether the H-UC-MSC treatment of SLE, which was successful in humans, could also cure the SLE-like phenotype in a SLE mouse model. Thus, we aimed to observe the effect of treating B6.Fas mice with H-UC-MSCs and the mechanism of action of H-UC-MSCs in the treatment of SLE.

MSCs are most abundant in bone marrow and umbilical cord blood [14]. Both UC-MSCs and BM-MSCs display stable expression of CD29, CD90, and CD13 but do not express CD34 or CD31 and do not express the costimulatory molecules CD80, CD86, and CD40. The expression of the integrin family member CD29, along with the adhesion molecules CD44 and CD105, is believed to be an important signature [15]. MSCs do not express MHC II molecules and do not express or express extremely low levels of MHC I molecules, consistent with the low immunogenicity of MSCs. These MHC II molecules mediate the human-induced immune rejection of allogeneic major antigens and account for the low immunogenicity of MSCs. Moreover, MSCs do not express the T cell surface molecules B7-1 or B7-2 or molecules stimulated during apoptosis and thus lack the necessary components for T cell activation.

H-UC-MSCs are pluripotent cells that can differentiate into various cell types, including bone, cartilage, fat, and muscle cells [16, 17]. Their immunomodulating effects suggest their potential application in treating SLE. BM-MSC transplantation can reverse SLE with multiple organ dysfunction in mice and humans [18]. Because treatment of SLE mice by H-UC-MSC transplantation has not been reported previously, we isolated and cultured H-UC-MSCs and transplanted these cells into B6.Fas mice for treatment. The results are reported below. This study elucidated the mechanism of action of H-UC-MSCs observed previously in humans, thus justifying the current work as a valuable complement to the studies in humans.

## 2. Materials and Methods

### 2.1. Experimental Materials, Reagents, Equipment, and Sources

C57BL/6 mice were purchased from the Experimental Animal Center of the Third Military Medical University. B6.Fas mice were purchased from Nanjing Experimental Animal Model Center. Antibodies were purchased from BD Biosciences. DiR iodide was purchased from Beijing Fan Bo Biological Technology Company, Ltd. Anti-nuclear and anti-histone antibody ELISA kits were purchased from Shanghai BlueGene Biotech Company. Anti-double-stranded DNA antibody ELISA kits were purchased from Wuhan Cusabio Biotechnology Company. Blood RNA extraction kits were purchased from Beijing Huayueyang Biotechnology Company, Ltd. Cellular RNA extraction kits and quantitative PCR reagents were purchased from Baitaike Company. The reverse transcription kits were purchased from Thermo Electron Corporation. The* in vivo* imager (IVIS Lumina XR) was manufactured by Caliper Life Sciences. All of the reagents were used before their expiration dates. Experimental protocols were approved by the Experimental Animal Ethics Committee of Kunming General Hospital of Chengdu Military Command.

### 2.2. Isolation and Culture of H-UC-MSCs

All participants provided written informed consent to participation in this study. All experiments were approved by the Human Research Ethics Committee of Kunming General Hospital of Chengdu Military Command that specifically considers studies in which umbilical cord samples are collected from human participants. Sterile umbilical cord specimens were obtained with informed consent from healthy mothers undergoing caesarean section, washed with saline, and soaked in double antibiotics. The capsule, umbilical artery, and umbilical vein were removed, and Wharton glial cells were cultured. When most of the bottom of the culture bottle was covered with cells, the medium (DMEM/F12 medium containing 20% FBS) was changed, and the cells were passaged.

### 2.3. Identification of H-UC-MSCs by Flow Cytometry

Third passage H-UC-MSCs were digested and divided into 3 Eppendorf (EP) tubes, with each tube containing 1 × 10^6^ cells. The first tube was labeled with CD29-FITC and CD34-PE, the second tube was labeled with CD31-FITC and CD90-PE, and the third tube was labeled with CD13-PE. FITC and PE isotype controls were used for FACS analysis. All the antibodies used in FACS analysis were purchased from BD Company. The cells were centrifuged, and the supernatant was discarded, followed by the addition of 50 *μ*L of FB (PBS + 2% fetal bovine serum + 0.1% NaN_3_). Then, 3 *μ*L of labeled antibody was added to each tube, followed by incubation at room temperature for 1 hour, 2 washes in FB, the addition of 400 *μ*L of fixative, and flow cytometric analysis (from BD, FACS Vantage SE).

### 2.4. Mouse Grouping

The following five groups were established: normal control (C57BL/6 mice) group; model (B6.Fas mice) group; and low-, medium-, and high-dose treatment (B6.Fas mice) groups. Each group consisted of 10 mice. The mice were all female and 8 weeks old.

### 2.5. H-UC-MSC Transplantation

H-UC-MSCs were transplanted through the tail vein. In total, 2 × 10^6^ cells were transplanted into each mouse in the B6.Fas mouse high-dose group, 1 × 10^6^ cells were transplanted into each mouse in the B6.Fas mouse medium-dose group, and 0.5 × 10^6^ cells were transplanted into each mouse in the B6.Fas mouse low-dose group. The B6.Fas mouse model group and the C57BL/6 mouse normal control group were not treated. Treatment was administered once per week for 4 weeks. At the end of treatment, blood samples were tested for anti-nuclear, anti-histone, and anti-double-stranded DNA antibodies. Blood RNA was analyzed to detect changes in gene expression.* Ex vivo* body imaging, kidney H&E staining, and Masson staining were performed.

### 2.6. DiR Cell Labeling

H-UC-MSCs were digested with 0.25% trypsin. Trypsinization was stopped by adding complete medium containing 20% FBS. Then, 5 *μ*L of 3 mM DiR storage solution was added to 1 mL of cell suspension containing 1 × 10^6^ cells/mL, followed by incubation at 37°C for 10 minutes and 3 washes in preheated serum-free medium (centrifugal rotation: 1500 r/min, centrifugation time: 5 minutes). Mice in the B6.Fas mouse high-dose group were injected with 2 × 10^6^ labeled cells, mice in the B6.Fas mouse medium-dose group were injected with 1 × 10^6^ labeled cells, and mice in the B6.Fas mouse low-dose group were injected with 0.5 × 10^6^ labeled cells. Treatment was administered once per week for 4 weeks. DiR-labeled H-UC-MSCs were used for the tail vein infusions.

### 2.7. Detection of Anti-Nuclear, Anti-Histone, and Anti-Double-Stranded DNA Antibodies

ELISA assays were performed on serum samples according to the ELISA test kit instructions.

### 2.8. Quantitative PCR Detection of OPG and Foxp3 Expression in Mouse Peripheral Blood

RNA was extracted from mouse tail blood according to the instructions provided with the Beijing Huayueyang Biotechnology Co., Ltd., kit. Using a reverse transcription kit, RNA was reverse transcribed into cDNA, and quantitative PCR was subsequently performed to detect changes in OPG and Foxp3 gene expression. RT-PCR was performed with 1 *μ*g of total RNA using a cDNA synthesis kit (Thermo). cDNA (20 *μ*L) was diluted 1 : 5, and 3 *μ*L was used in each reaction of triplicate quantitative PCRs on a real-time PCR detection system with IQ SYBR Green (Bio-Rad). The primers used are listed in Table 1. The PCR conditions were as follows: 95°C for 3 min and 40 cycles of 95°C for 30 s, 60°C for 30 s, and 72°C for 30 s. The data were analyzed using formulas from Pfaffl, 2001, with glyceraldehyde-3-phosphate dehydrogenase (GAPDH) as the normalization control.

### 2.9. Testing for Murine Regulatory T Cells in Peripheral Blood

The number of murine regulatory T cells in peripheral blood decreases during SLE disease progression. Tail blood was collected from mice in each group, and 50 *μ*L of anticoagulant was added to each sample. Then, 0.5 *μ*L of CD4-FITC and 0.5 *μ*L of CD25-PE were added to 100 *μ*L samples of anticoagulated blood, and the samples were incubated at room temperature in the dark for 30 min, followed by the addition of 1.5 mL of fixation/permeabilization solution and incubation at room temperature for 20 min. Then, the cells were centrifuged at 2000 rpm for 5 min, and the supernatant was discarded. Next, 100 *μ*L of staining buffer was added along with 2.5 *μ*L of Foxp3-PE-Cy5. The samples were incubated in the dark at room temperature for 30 min and analyzed by flow cytometry.

### 2.10. Detection of 24-Hour Urine Protein Levels

At the end of treatment, each mouse was placed in a metabolic cage, and 24-hour urine samples were collected. Urine volumes were determined, and urine protein was detected using the Bradford method. The 24-hour urine proteins were compared among the five groups.

### 2.11. Analysis of the Distribution of H-UC-MSCs in Various Organs by* Ex Vivo* Imaging

B6.Fas mice were injected with transplanted cells though the tail vein once every week for four weeks, and the mice were sacrificed at two weeks after the end of treatment. DiR-labeled cells were observed using* ex vivo* imaging to assess the distribution of these cells to various organs.

### 2.12. Pathological Analysis of Various Organ Lesions in Each Group

Isolated organs were immersed in 4% paraformaldehyde after* ex vivo* imaging and sent to Google Biotechnology Co., Ltd., for paraffin sectioning, H&E staining, and Masson staining of the kidney. The deposition of immune complexes in the kidneys was detected by PE-labeled goat anti-mouse IgG and observed using a fluorescence microscope.

### 2.13. Statistical Analysis

The data values are shown as the mean ± SD. Groups were compared by one-way ANOVA using SPSS 17.0 statistical software. *P* < 0.05 was considered statistically significant.

## 3. Results

### 3.1. Morphology and Identification of H-UC-MSCs

**Figure 1:**
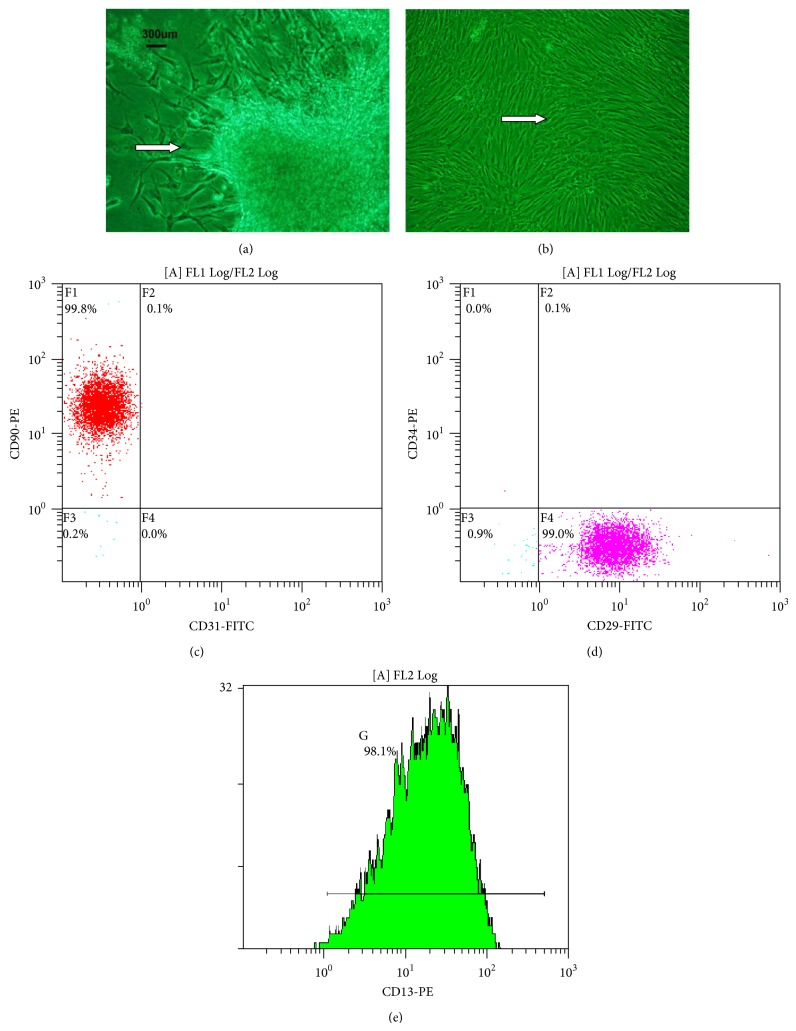
H-UC-MSC morphology. (a) Cells after 7 days in culture. (b) Third passage cells in culture H-UC-MSC flow cytometry results. (c) CD90-PE and CD31-FITC double labeling. (d) CD34-PE and CD29-FITC double labeling. (e) CD13-PE single labeling. The arrows show H-UC-MSCs.

H-UC-MSCs were cultured for seven days, and the resulting adherent cells exhibited fusiform growth (Figure 1(a)). When these cultures reached the third passage, the visible growth of adherent cells exhibited a uniform fusiform distribution (Figure 1(b)). Because we used phase contrast microscopy to observe the cells, the cells appear green.

### 3.2. Identification of H-UC-MSCs by Flow Cytometry

Because H-UC-MSCs strongly express CD90, CD29, and CD13 and do not express the hematopoietic cell marker CD34 or the endothelial cell marker CD31, these five antibodies were used to detect H-UC-MSCs. The cells were strongly positive for CD90, CD29, and CD13 expression and negative for CD34 and CD31 expression, indicating that our isolated and cultured H-UC-MSCs are of high purity. Flow cytometric results showed that the H-UC-MSCs expressed CD90, CD29, and CD13 but did not express CD31 or CD34, indicating that the isolated H-UC-MSCs were of high purity (Figures 1(c)–1(e)).

### 3.3. Analysis of Anti-Nuclear, Anti-Histone, and Anti-Double-Stranded DNA Antibodies in the C57BL/6 Mouse Normal Control Group, the B6.Fas Mouse Model Group, and the Three B6.Fas Mouse Treatment Groups after Treatment

**Figure 2:**
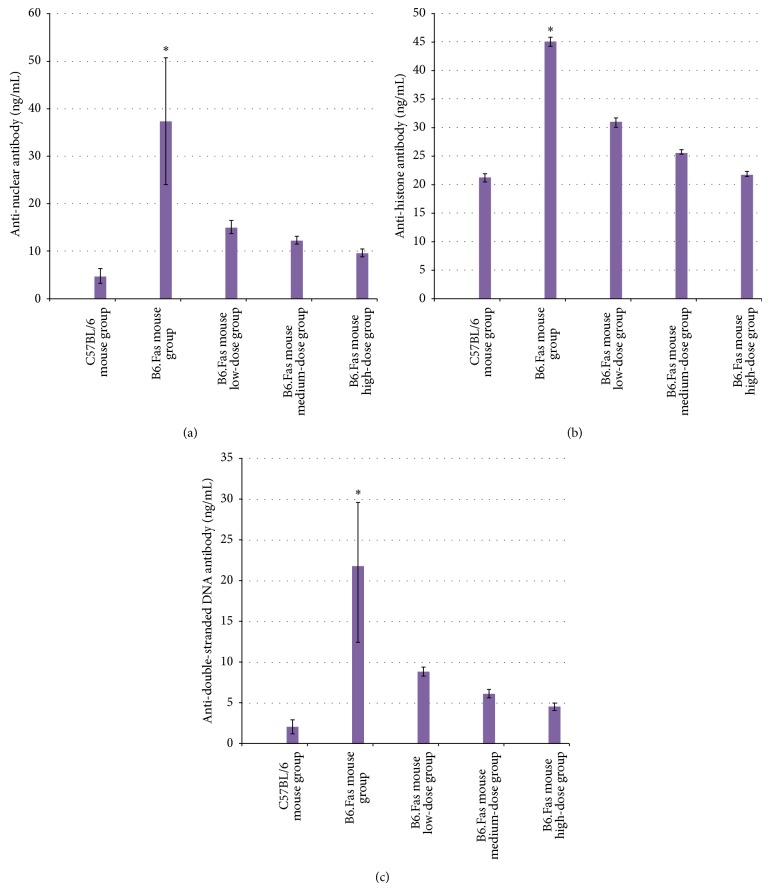
(a) Anti-nuclear antibody testing in the five groups. The results are expressed as the mean ± standard deviation (*n* = 10). (b) Anti-histone antibody testing in the five groups. The results are expressed as the mean ± standard deviation (*n* = 10). (c) Anti-double-stranded DNA antibody testing in the five groups. The results are expressed as the mean ± standard deviation (*n* = 10). ^*^ *P* < 0.01 compared to other groups.

The results of anti-nuclear, anti-histone, and anti-double-stranded DNA antibody testing for the five groups are shown in Figure 2. The B6.Fas mouse model group displayed significantly higher levels of anti-nuclear, anti-histone, and anti-double-stranded DNA antibodies than those of the B6.Fas mouse treatment groups.

The results of the anti-nuclear, anti-histone, and anti-double-stranded DNA antibody analysis demonstrated statistically significant differences among the groups (*P* < 0.01).

#### 3.3.1. Analysis of Anti-Nuclear Antibodies

A pairwise comparison of the five groups revealed values of *P* < 0.01 for the C57BL/6 mouse normal control group and the B6.Fas mouse model group and of *P* < 0.01 for the B6.Fas mouse model group compared with the other four groups, but it revealed a value of *P* = 0.083 for the C57BL/6 mouse normal control group compared with the B6.Fas mouse high-dose group. This result indicated that the anti-nuclear antibody levels in the B6.Fas mouse high-dose group were close to those of the C57BL/6 mouse normal control group after treatment.

#### 3.3.2. Analysis of Anti-Histone Antibodies

A pairwise comparison of the five groups revealed values of *P* < 0.01 for the C57BL/6 mouse normal control group and the B6.Fas mouse model group and of *P* < 0.01 for the B6.Fas mouse treatment groups compared with the B6.Fas mouse model group. However, it revealed a value of *P* = 0.043 for the C57BL/6 mouse normal control group compared with the B6.Fas mouse high-dose group. This result indicated that anti-histone antibody levels decreased in the B6.Fas mouse high-dose group after treatment and that this difference from the C57BL/6 mouse normal control group was statistically significant.

#### 3.3.3. Analysis of Anti-Double-Stranded DNA Antibodies

A pairwise comparison of the five groups revealed values of *P* < 0.01 between the C57BL/6 mouse normal control group and the B6.Fas mouse model group and of *P* < 0.01 for the B6.Fas mouse model group compared with the other four groups. However, it revealed a value of *P* = 0.163 for the C57BL/6 mouse normal control group compared with the B6.Fas mouse high-dose group, explaining the similar results regarding anti-double-stranded DNA antibodies between the B6.Fas mice high-dose group and the C57BL/6 mouse normal control group.

The transplanted H-UC-MSCs improved the autoantibody levels most likely by inducing the clearance of the autoantibodies or by affecting the apoptosis defects described in the SLE model.

### 3.4. Comparison of H&E Staining between the C57BL/6 Mouse Normal Control Group, the B6.Fas Mouse Model Group, and the Three B6.Fas Mouse Treatment Groups after Treatment

#### 3.4.1. Spleen H&E Staining (Figures 3(a)–3(e))

The B6.Fas mouse model group displayed visible hemosiderin, indicating megakaryocytes. The arrow indicates an area with megakaryocytes.

#### 3.4.2. Liver H&E Staining (Figures 3(f)-3(j))

The B6.Fas mouse model group presented visible spots, indicating liver cell necrosis. The arrow indicates an area of necrotic liver cells.

#### 3.4.3. Kidney H&E Staining (Figures 3(k)–3(o))

The B6.Fas mouse model group presented slightly proliferative mesangial cells along with reduced renal cysts. The arrow shows the reduced renal cysts.

#### 3.4.4. Lung H&E Staining (Figures 3(p)–3(t))

The B6.Fas mouse model group presented visible alveolar septal infiltration of inflammatory cells. The arrow indicates inflammatory cell infiltration.

#### 3.4.5. Masson Staining of the Kidney in the C57BL/6 Mouse Normal Control Group, the B6.Fas Mouse Model Group, and the Three B6.Fas Mouse Treatment Groups after Treatment (Figures 3(u)–3(y))

The B6.Fas mice displayed slight fibrosis of the tubulointerstitial renal cortex. The arrow indicates fibrosis of the tubulointerstitial renal cortex.

The abnormal conditions observed in spleen, liver, kidney, lung, and kidney by Masson staining appeared to improve in the three treatment groups.

#### 3.4.6. The Deposition of Immune Complexes in the Kidneys as Observed by Fluorescence Microscopy (Figure 4)

**Figure 4:**
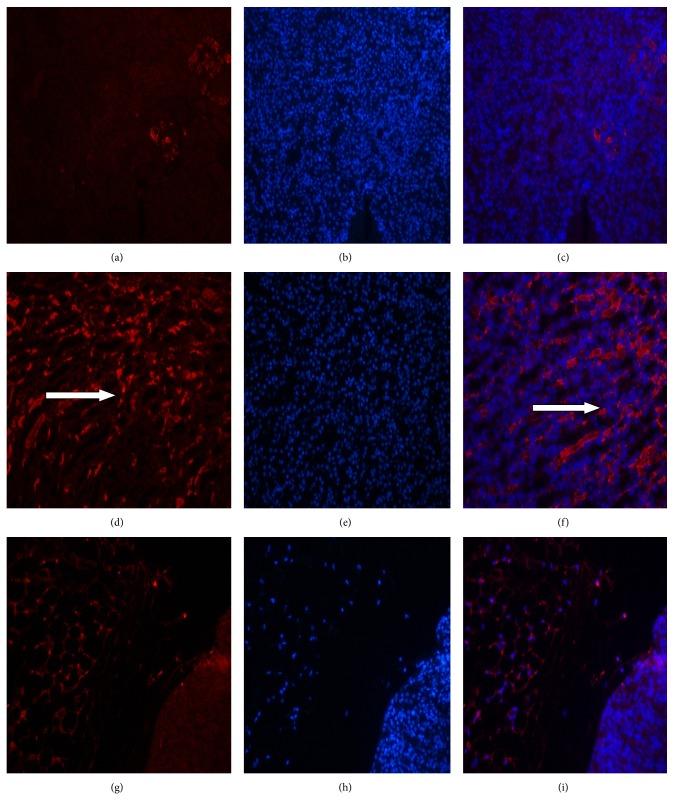
The deposition of immune complexes in the kidneys as observed by fluorescence microscopy. (a–c) C57BL/6 mouse normal control group. (d–f) B6.Fas mouse model group. (g–i) B6.Fas mouse high-dose group. ((a), (d), and (g)) PE staining. ((b), (e), and (h)) DAPI staining. ((c), (f), and (i)) Merged images. The arrows show red fluorescence immune complexes.

Red fluorescent immune complexes were observed in the kidneys of mice in the B6.Fas mouse model group (Figures 4(d) and 4(f)), and few red fluorescent immune complexes were observed in the kidneys of mice in the control group (Figures 4(a) and 4(c)). Red fluorescent immune complexes were decreased in the kidneys of mice in the B6.Fas mouse high-dose group compared to those of mice in the C57BL/6 mouse normal control group (Figures 4(g) and 4(i)), thus indicating that some immune complexes in the kidneys had been cleared after treatment in the B6.Fas mouse high-dose group. Red fluorescence indicates immune complexes.

### 3.5. PCR-Based Quantitative Detection of Changes in OPG and Foxp3 RNA Expression in Peripheral Blood Samples from the Five Groups after Treatment

**Figure 5:**
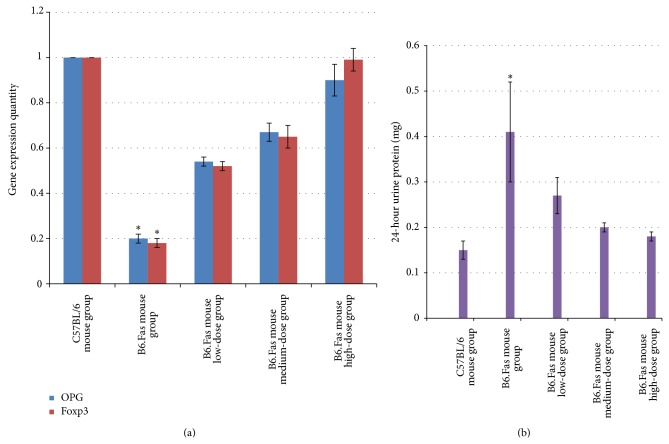
(a) Results of quantitative PCR tests for two genes in each group. ^*^ *P* < 0.01 compared to other groups. (b) Comparison of 24-hour urine protein levels among the groups. The values are expressed as the mean ± standard deviation (*n* = 10).

As observed in Figure 5(a), the levels of OPG and Foxp3 expression decreased in peripheral blood samples from the B6.Fas mouse model group, which is consistent with other results in the literature. After treatment, the OPG and Foxp3 expression levels returned to normal.

The changes in gene expression were significantly different among the groups (*P* < 0.01).

The multiple comparison results indicated that the differences between any two groups were statistically significant (*P* < 0.01).

### 3.6. Twenty-Four-Hour Urine Protein Levels in Each Group

As observed in Figure 5(b), the 24-hour urine protein levels in the B6.Fas mouse model group were significantly higher than in the C57BL/6 mouse normal control group or in the three B6.Fas mouse treatment groups, indicating decreased renal function in the B6.Fas mouse model group.

The results of the 24-hour urine protein analysis for each group were statistically significant (*P* < 0.01).

Pairwise comparisons indicated values of *P* < 0.01 for the C57BL/6 mouse normal control group and the B6.Fas mouse model group and of *P* < 0.01 for the B6.Fas mouse treatment groups compared with the B6.Fas mouse model group. However, pairwise comparisons indicated values of *P* = 0.552 for the C57BL/6 mouse normal control group compared with the B6.Fas mouse high-dose group and of *P* = 0.248 for the C57BL/6 mouse normal control group compared to the B6.Fas mouse medium-dose group. This result indicated that the urine protein levels in the B6.Fas mouse high-dose and medium-dose groups decreased to near those of the C57BL/6 mouse normal control group.

### 3.7. Results of Flow Cytometry for CD4^+^CD25^+^Foxp3^+^ Cells in the C57BL/6 Mouse Normal Control Group, the B6.Fas Mouse Model Group, and the Three B6.Fas Mouse Treatment Groups after Treatment

**Figure 6:**
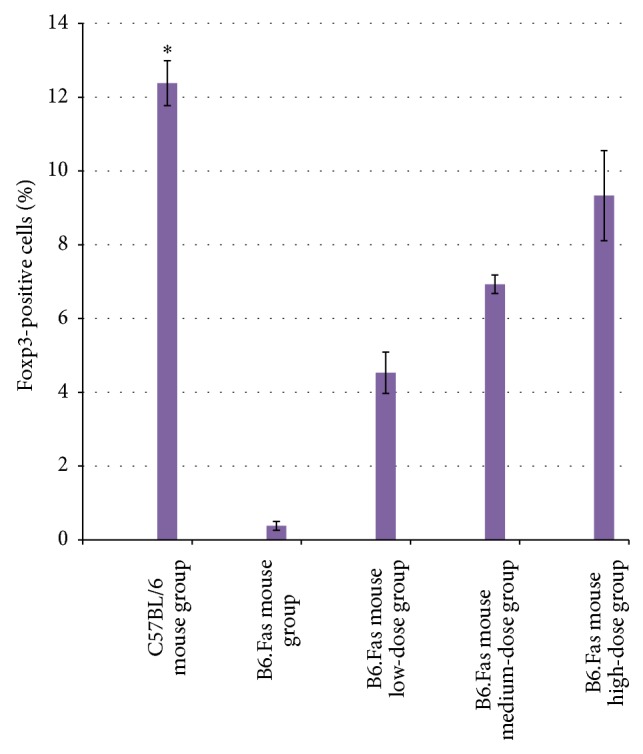
Percentages of Foxp3-positive cells in the five groups. The percentage of Foxp3-positive cells significantly decreased in the B6.Fas mouse model group compared with that of the C57BL/6 mouse normal control group. After treatment, the percentages of Foxp3-positive cells increased in the low-, medium-, and high-dose groups. The data are expressed as the mean ± standard deviation (*n* = 10). ^*^ *P* < 0.01 compared to other groups.

The results show that the number of CD4^+^CD25^+^Foxp3^+^ cells significantly decreased in the B6.Fas mouse model group compared to that in the C57BL/6 mouse normal control group. The percentage of CD4^+^CD25^+^Foxp3^+^ cells increased after treatment, and the percentage of cells in the B6.Fas mouse high-dose group increased to near that of the C57BL/6 mouse normal control group (Figure 6).

The flow cytometry analysis results for CD4^+^CD25^+^Foxp3^+^ cells were statistically significant among the groups (*P* < 0.01).

A pairwise comparison among the five groups revealed a value of *P* < 0.01 between any two groups.

### 3.8. After Treatment, Mouse Organs Were Processed for* Ex Vivo* Imaging

#### 3.8.1. Cell Labeling

**Figure 7:**
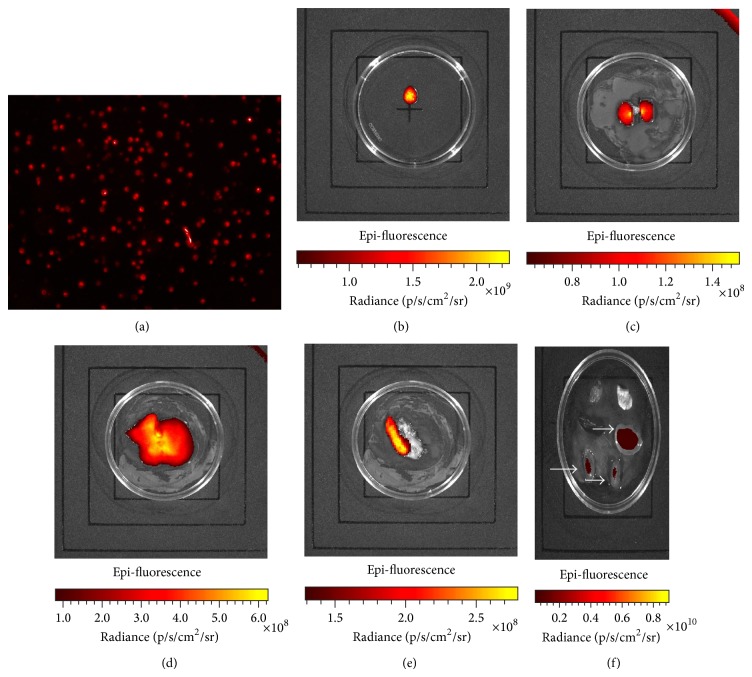
Cells were labeled with a dark red fluorescent marker. (a) Fluorescence microscopy images. (b)* Ex vivo* image observations. (c–f)* Ex vivo* imaging results of various organs from the treatment groups. (c) Labeled cells distributed in the kidney. (d) Labeled cells distributed in the liver. (e) Labeled cells distributed in the spleen and pancreas. (f) Observation of labeled cells in the kidneys, spleen, liver, lung, and heart. Arrows show aggregated DiR-stained cells in the liver and two kidneys.

Labeled cells were monitored by fluorescence microscopy (Figure 7(a)). The labeled cells were processed for* ex vivo* imaging as follows: a 0.1 mL cell suspension containing 1 × 10^5^ cells was observed on plates using an* ex vivo* imager, with labeled cells displaying visible dark red fluorescence (Figure 7(b)).

#### 3.8.2. *Ex Vivo* Imaging of the Kidney, Liver, and Spleen

DiR-labeled H-UC-MSCs were infused into SLE mice, and organs were harvested for* ex vivo* imaging at 2 weeks after infusion. Aggregation of labeled cells was observed in the kidney (Figure 7(c)), indicating that the cells were involved in the repair of renal lesions. Some labeled cells were also observed in the liver (Figure 7(d)), demonstrating the retention and incorporation of some cells into that organ. Some labeled cells were also found in the spleen (Figure 7(e)), although no labeled cells were observed in other organs. No further distribution of labeled cells was observed in the C57BL/6 mouse normal control group and in the B6.Fas mouse model group, indicating that cells primarily accumulated in the liver and kidney after intravenous transfusion (Figure 7(f)). Because B6.Fas mouse model displays nephritis typical of SLE, transplanted H-UC-MSCs will translocate to the kidneys to participate in kidney damage repair.

## 4. Discussion

Clinically, BM-MSCs have been successfully utilized to treat a variety of human injuries and diseases, including bone fractures [19], severe aplastic anemia [20], and acute GVHD [21]. Thus, BMMSCs are a promising population of postnatal stem cells for use in clinical therapies. In this study, we used H-UC-MSCs to treat B6.Fas mice and observed the efficacy and mechanism of H-UC-MSC therapy. H-UC-MSCs were cultured and purified in the present study. The isolated cells were positive for CD29 and CD90 expression and were negative for CD31 and CD34 expression. Positive CD13 expression indicated that the isolation and culture of H-UC-MSCs were successful.

Experimental model animals were purchased to generate more accurate and reliable results. B6.Fas mice display hallmarks of autoimmune disease [22], including increased levels of anti-nuclear, anti-histone, and anti-double-stranded DNA antibodies, as well as pathological changes to the spleen, liver, and kidneys. According to the literature [23], gene expression in B6.Fas mice is altered as follows: OPG and Foxp3 gene expression decrease, and RANKL and IL-17 gene expression increase. We extracted RNA from collected blood samples and observed that OPG and Foxp3 expression indeed changed compared with those of the C57BL/6 mouse normal control group. This result indicated that our mouse model was reliable.

Foxp3-positive T cells were detected in peripheral blood at two weeks after treatment. The results showed that the number of Foxp3-positive T cells decreased significantly in the B6.Fas mouse model group compared with the C57BL/6 mouse normal control group, suggesting decreased numbers of immune cells in the B6.Fas mouse model. After treatment, the numbers of Foxp3-positive T cells increased in the B6.Fas mouse low-, medium-, and high-dose groups. Thus, immune regulation was restored, and the immune cell levels better matched those in the C57BL/6 mouse normal control group. Other studies have demonstrated higher numbers of CD4^+^CD25 (high) FoxP3^+^ cells in SLE patients than in healthy donors [24–26]. In contrast, our SLE model exhibits low numbers of this cell type, and the numbers of these cells increase after H-UC-MSC treatment. Thus, T and B lymphocyte abnormalities are universal among SLE patients. The numbers of CD4^+^CD25 (high) cells decreased in the SLE model.

Our experiments used H-UC-MSCs labeled with deep red fluorescent DiR. These cells were infused into mice, and the mice were sacrificed for* ex vivo* imaging after treatment. Sacrifice of the animals was necessary to observe the distribution of labeled cells more clearly in various organs than is possible with* in vivo* imaging of whole mice. The results showed that labeled cells were distributed in the kidney, liver, and spleen, although the specific mechanism remained unclear. Because B6.Fas mice display nephritis typical of SLE, transplanted H-UC-MSCs will translocate to the kidneys to participate in kidney damage repair. Cues that attract or direct H-UC-MSCs must be present. H-UC-MSCs were also distributed in the liver and spleen, further illustrating the ability of these cells to reach multiple organs and to repair multiple organ injuries. Histological analysis demonstrated that the livers and spleens of B6.Fas mice also display pathological injuries, and the labeled cells colonized and repaired these injuries after treatment.

In summary, H-UC-MSCs have a multipotent differentiation capacity and may play therapeutic roles through the regulation of lymphocyte function, induction of immune tolerance, and suppression of the autoimmune response [27]. H-UC-MSCs can be derived from the umbilical cord and placenta. H-UC-MSCs have many advantages. H-UC-MSCs from umbilical cord tissue are pluripotent stem cells, can be amplified easily, are available for repeated use, and can be used for the treatment of tissue and organ damage and of systemic diseases of the nervous system, immune system, endocrine system, and other systems. This treatment is available for people of all ages. Conversely, UC-HSCs from umbilical cord blood are lineage-restricted stem cells, are difficult to amplify* in vitro*, are typically obtained for a single use, and are primarily used to treat blood diseases, such as leukemia, aplastic anemia, and Mediterranean anemia. The clinical use of H-UC-MSCs is increasingly being reported due to the wide variety of potential sources, the ease with which the cells can be obtained, the lack of ethical issues, and the lack of adverse effects to the donor. Additionally, H-UC-MSCs display low immunogenicity, do not result in GVHD, and are suitable for large-scale cultivation* in vitro*. H-UC-MSC transplantation for the treatment of SLE is relatively new; however, this application has broad prospects for cell therapy. As shown in recent studies [28], the application of H-UC-MSCs for the treatment of severe, recurrent SLE resulted in a significant decrease in the patient's disease activity index. Serum anti-nuclear and anti-dsDNA antibody concentrations decreased, albumin and C3 levels improved, and renal function recovered. No transplant-related complications occurred, and the number of Treg cells increased after treatment. H-UC-MSC transplantation in B6.Fas mice was described in the present study. The data show great improvement in the SLE symptoms generated in B6.Fas mice. With increased understanding of this new technology, these potential clinical applications will continue to develop, improving the prospects for patients with SLE.

## 5. Conclusions

In summary, our study found that B6.Fas mice display various hallmarks of SLE, including increased anti-nuclear, anti-histone, and anti-double-stranded DNA antibodies; liver, kidney, and spleen diseases; changes in OPG and Foxp3 gene expression in the peripheral blood; and changes in the numbers of regulatory T cells. We treated B6.Fas mice with up to four rounds of H-UC-MSC treatment and observed statistically significant improvement of disease symptoms compared with the model group. Because these H-UC-MSCs can be easily obtained from a wide variety of sources without adversely affecting the donor, display low immunogenicity, are not limited by ethical issues, do not elicit GVHD, display superior proliferation and differentiation abilities, and are suitable for large-scale cultivation* in vitro*, these provide a new cell therapy for SLE disease.

## Figures and Tables

**Figure 3:**
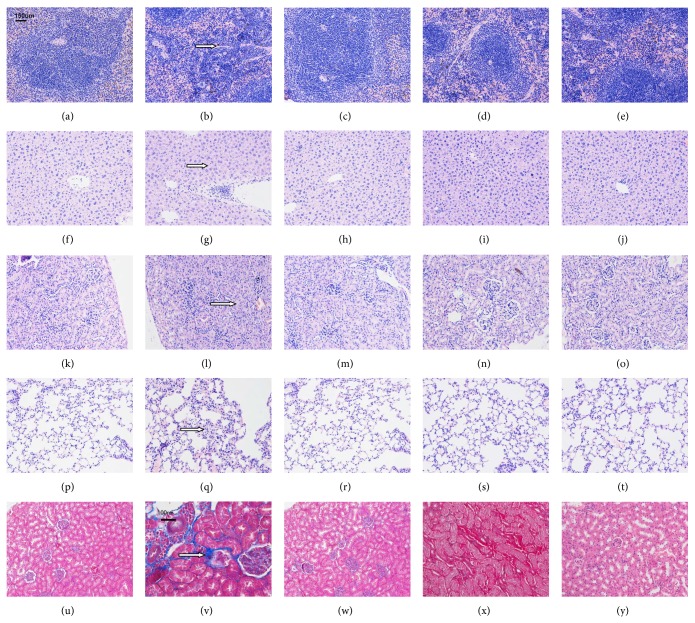
(a–e) Spleen H&E staining (200x magnification). (a) C57BL/6 mouse normal control group. (b) B6.Fas mouse model group. The arrow indicates megakaryocytes. (c) B6.Fas mouse low-dose group. (d) B6.Fas mouse medium-dose group. (e) B6.Fas mouse high-dose group. The B6.Fas mouse model group displayed visible hemosiderin staining, which improved after treatment. (f–j) Liver H&E staining (200x magnification). (f) C57BL/6 mouse normal control group. (g) B6.Fas mouse model group. The arrow shows necrotic liver cells. (h) B6.Fas mouse low-dose group. (i) B6.Fas mouse medium-dose group. (j) B6.Fas mouse high-dose group. The B6.Fas mouse model group displayed visible spots of necrotic liver cells, which improved after treatment. (k–o) Kidney H&E staining (200x magnification). (k) C57BL/6 mouse normal control group. (l) B6.Fas mouse model group. The arrow shows reduced renal cysts. (m) B6.Fas mouse low-dose group. (n) B6.Fas mouse medium-dose group. (o) B6.Fas mouse high-dose group. The B6.Fas mouse model group displayed slightly proliferative mesangial cells and reduced renal cysts, which improved after treatment. (p–t) Lung H&E staining (200x magnification). (p) C57BL/6 mouse normal control group. (q) B6.Fas mouse model group. The arrow shows inflammatory cell infiltration. B6.Fas mouse low-dose group. (s) B6.Fas mouse medium-dose group. (t) B6.Fas mouse high-dose group. The B6.Fas mouse model group displayed visible alveolar septal infiltration of inflammatory cells, which improved after treatment. (u–y) Renal Masson staining. (u) C57BL/6 mouse normal control group (200x magnification). (v) B6.Fas mouse model group (400x magnification). The arrow shows fibrosis of the tubulointerstitial renal cortex. (w) B6.Fas mouse low-dose group (200x magnification). (x) B6.Fas mouse medium-dose group (400x magnification). (y) B6.Fas mouse high-dose group (200x magnification). The B6.Fas mouse model group displayed slight fibrosis of the tubulointerstitial renal cortex, which improved after treatment. Arrows show the abnormal area.

**Table 1:** Primer sequences.

	Primer sequence	Product length (bp)
OPG	5′-ATCAGAGCCTCATCACCTT-3′	181
	5′-CTTAGGTCCAACTACAGAGGAAC-3′	

Foxp3	5′-CCCAGGAAAGACAGCAACCTT-3′	160
	5′-CCTTGCCTTTCTCATCCAGGA-3′	

GAPDH	5′-CACCATGGAGAAGGCCGGGGG-3′	418
	5′-GACGGACACATTGGGGGTAG-3′	
